# Effect of elevated fasting blood glucose level on the 1‐year mortality and sequelae in hospitalized COVID‐19 patients: A bidirectional cohort study

**DOI:** 10.1002/jmv.27737

**Published:** 2022-04-19

**Authors:** Chen Chai, Kui Chen, Shoupeng Li, Gang Cheng, Wendan Wang, Hongxiang Wang, Dunshuang Wei, Cao Peng, Qi Sun, Zehai Tang

**Affiliations:** ^1^ Department of Emergency Medicine, Union Hospital, Tongji Medical College Huazhong University of Science and Technology Wuhan China; ^2^ Department of Emergency Medicine Zhongnan Hospital of Wuhan University Wuhan China; ^3^ Department of Emergency Medicine, Affiliated Dongfeng Hospital Hubei University of Medicine Shiyan Hubei China; ^4^ Department of Emergency Medicine, Huanan Hospital Shenzhen University Shenzhen China; ^5^ Computer Center, Union Hospital, Tongji Medical College Huazhong University of Science and Technology Wuhan China; ^6^ Department of Hematology, The Central Hospital of Wuhan, Tongji Medical College Huazhong University of Science and Technology Wuhan China; ^7^ Department of Emergency Medicine The Third People's Hospital of Hubei Province Wuhan China

**Keywords:** 1‐year mortality, clinical sequelae, coronavirus disease 2019, fasting blood glucose, nondiabetics

## Abstract

To observe the predictive effect of fasting blood glucose (FBG) level on the prognosis, clinical sequelae, and pulmonary absorption in hospitalized coronavirus disease 2019 (COVID‐19) patients with and without a history of diabetes, respectively, and to evaluate the correlation between the dynamic changes of FBG and poor prognosis. In this bidirectional cohort study, we enrolled 2545 hospitalized COVID‐19 patients (439 diabetics and 2106 without a diabetic history) and followed up for 1 year. The patients were divided according to the level of admission FBG. The dynamic changes of FBG were compared between the survival and the death cases. The prediction effect of FBG on 1‐year mortality and sequelae was analyzed. The 1‐year all cause mortality rate and in‐hospital mortality rate of COVID‐19 patients were J‐curve correlated with FBG (*p* < 0.001 for both in the nondiabetic history group, *p* = 0.004 and *p* = 0.01 in the diabetic history group). FBG ≥ 7.0 mmol/L had a higher risk of developing sequelae (*p* = 0.025) and have slower recovery of abnormal lung scans (*p* < 0.001) in patients who denied a history of diabetes. Multivariable Cox regression analysis showed that FBG ≥ 7.0 mmol/L was an independent risk factor for the mortality of COVID‐19 regardless of the presence or deny a history of diabetes (hazard atio [HR] = 10.63, 95% confidence interval [CI]: 7.15−15.83, *p* < 0.001; HR = 3.9, 95% CI: 1.56−9.77, *p* = 0.004, respectively). Our study shows that FBG ≥ 7.0 mmol/L can be a predictive factor of 1‐year all‐cause mortality in COVID‐19 patients, independent of diabetes history. FBG ≥ 7.0 mmol/L has an advantage in predicting the severity, clinical sequelae, and pulmonary absorption in COVID‐19 patients without a history of diabetes. Early detection, timely treatment, and strict control of blood glucose when finding hyperglycemia in COVID‐19 patients (with or without diabetes) are critical for their prognosis.

## 1 INTRODUCTION

Coronavirus disease 2019 (COVID‐19), caused by severe acute respiratory syndrome coronavirus 2 (SARS‐CoV‐2), has posed a serious threat to global health.^1^ According to epidemiological and clinical data, patients with COVID‐19 have higher risks of elevated blood glucose levels. COVID‐19 patients with hyperglycemia will be more likely to transform into severe and fatal cases.^2^, ^3^, ^4^ Cai et al.^5^ demonstrated that COVID‐19 patients with fasting blood glucose (FBG) ≥7.0 mmol/L had poor prognoses. Huang et al. proved that FBG was a strong predictor for in‐hospital mortality in COVID‐19 patients.^6^

At present, many studies investigated the effect of FBG on the short‐term adverse prognosis in COVID‐19 patients, but most studies are limited by follow‐up time and sample size. In terms of the stratification of FBG levels in nondiabetic COVID‐19 patients, most research used dichotomy (<7.0 and ≥7.0 mmol/L), some used trichotomy (<6.1, 6.1−7.0, and ≥7.0 mmol/L),^2^ while few studies focused on further stratification of FBG.^7^ Based on the criteria of the American Diabetes Association (ADA) and World Health Organization (WHO),^8^, ^9^ FBG in the prediabetic status is defined as 5.6−6.9 and 6.1−6.9 mmol/L, respectively. Therefore, in this investigation, we divided admission FBG into four levels in COVID‐19 patients without a diabetic history (≤5.5, 5.6−6.0, 6.1−6.9, and ≥7.0 mmol/L) and three levels in COVID‐19 patients with a diabetic history (≤6.1, 6.1−6.9, and ≥7.0 mmol/L), so as to investigate the effect of FBG on 1‐year mortality, sequelae, and pulmonary absorption in hospitalized COVID‐19 patients with and without diabetic history, respectively. On the other hand, few studies report the dynamic changes of FBG in COVID‐19 patients during hospitalization and its predictive effect on their long‐term prognosis.^9^ To observe the impact of SARS‐CoV‐2 infection on FBG and avoid the possible confounding factors of diabetes on FBG, we divided COVID‐19 patients into the diabetic history group and nondiabetic history group, analyzed the dynamic changes of FBG in patients with different admission FBG levels, and compared the level of FBG in the survival and death cases to study the impact of FBG level on the long‐term prognosis in the two groups.

## 2 METHODS

### 2.1 Study design and participants

**Figure 1:**
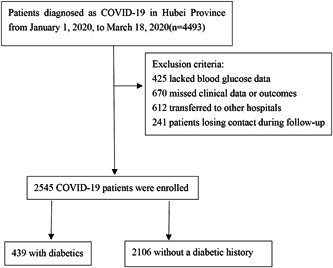
Flow chart of patient selection. We enrolled 439 coronavirus diseases 2019 (COVID‐19) patients with a diabetic history and 2106 COVID‐19 patients without a diabetic history

In this bidirectional cohort study, we chose 4493 hospitalized COVID‐19 patients who were admitted to five designated COVID‐19 hospitals from January 1, 2020 to March 18, 2020 in Hubei Province, including the headquarter, west Hospital, and tumor center of Union Hospital, the Central Hospital of Wuhan, and Dongfeng Hospital.^10^ Among the patients, 425 lacked blood glucose data, 670 missed clinical data or outcomes, 612 transferred to other hospitals, and 241 patients losing contact during follow‐up were excluded. Therefore, a total of 2545 COVID‐19 patients (439 diabetics and 2106 without a diabetic history) were enrolled (Figure 1). All patients met the following conditions: (1) positive reverse transcription‐polymerase chain reaction (RT‐PCR) detection of SARS‐CoV‐2 in respiratory tract samples; (2) clinically confirmation of COVID‐19; (3) complete and traceable clinical information; (4) complete follow‐up and answer of questionnaires. According to the FBG level at admission, COVID‐19 patients without a diabetic history were divided into four groups: FBG ≤ 5.5, 5.6−6.0, 6.1−6.9, and ≥7.0 mmol/L,^8^ and COVID‐19 patients with a diabetic history were divided into three groups: FBG ≤ 6.1 6.1−6.9, and ≥7.0 mmol/L.

### 2.2 Data collection

All data from the electronic medical records of the 2545 COVID‐19 patients were reviewed manually. The demographic characteristics, comorbidities, clinical signs and symptoms, laboratory results, in‐hospital treatment (including drug and oxygen therapy), and clinical outcome (discharge or death) were recorded using standardized case report forms and entered into a secure online database MySQL.^10^

### 2.3 Case follow‐up

All survival discharged patients underwent a series of telephone questionnaires or outpatient services every 3 months, including all‐cause death, clinical sequelae, and lung condition. Death and its cause were reported by the patient's relative or clinician. The follow‐up time was up to March 17, 2021, or the date of death.

### 2.4 Statistical analysis

All data were analyzed by SPSS Statistics 26.0 (IBM), GraphPad Prism 8.0.2 (Version 5.0; GraphPad Software), and R software (Version 3.6.3; Math Soft). Descriptive statistics were used for baseline data, categorical variables were expressed as proportions, and the normally distributed measurement data were shown as mean ± standard deviation (SD). One‐way analysis of variance (ANOVA) was used for intergroup comparison. Least significant difference *t*‐test was used for homogeneity of variance and Tamhane T2 test was used for heterogeneity of variance in pairwise comparison. The non‐normally distributed measurement data were shown as median (interquartile range, IQR). The nonparametric Kruskal−Wallis *H* test was used for intergroup comparison. The Mann−Whitney *U* test was used for pairwise comparison. Fisher's exact test was used for counting data. The model of the dynamic trajectory of FBG was established using Lowess. Kaplan−Meier and log‐rank tests were used to compare the survival curves of the nondiabetic patients. Cox regression was used to analyze the influence of several risk factors on survival rate. The difference was considered to be statistically significant at *p* < 0.05.

## 3 RESULTS

### 3.1 Patient characteristics

A total of 2545 hospitalized COVID‐19 patients (439 diabetics and 2106 without a diabetic history) were included in this study (Table 1). Among the 439 COVID‐19 patients with a diabetic history, the cases of admission FBG < 6.1, 6.1−6.9, and ≥7.0 mmol/L were 91 (20.7%), 58 (13.2%), and 290 (66.1%), respectively. The distribution of FBG in diabetics was not related to the age and gender of patients and showed no significant difference in severity, comorbidities, clinical symptoms, complications, and specialized treatment of COVID‐19. Among the 2106 COVID‐19 patients without a diabetic history, the cases of admission FBG ≤ 5.5, 5.6−6.0, 6.1−6.9, and ≥7.0 mmol/L were 1107 (52.6%), 446 (21.2%), 253 (12.0%), and 300 (14.2%), respectively, with 17 (0.8%) in FBG < 3.9 mmol/L (Table 1). Patients with admission FBG ≥ 7.0 mmol/L tended to be elder (>65 years old), male, and severe (all *p* < 0.001). In terms of clinical symptoms, patients with FBG ≥ 7.0 mmol/L were more prone to having fatigue, expectoration, dyspnea, and consciousness disorder (all *p* < 0.001). Regarding comorbidities, FBG levels were higher in patients with cancer (*p* = 0.047). As for complications, patients with FBG ≥ 7.0 mmol/L had increased rates of hypoproteinemia, respiratory failure, acute respiratory distress syndrome, acute kidney injury, acute liver injury, and lower limb venous thrombosis (all *p* < 0.001). And for treatment, patients with FBG ≥ 7.0 mmol/L were more likely to be treated with antibiotics, glucocorticoids, vasoactive drugs, and mechanical ventilation (all *p* < 0.001) (Table 1).

**Table 1:** Demographic and clinical data of 2545 hospitalized COVID‐19 patients

	Denied diabetes					Diabetes			
Characteristics	FBG ≤ 5.5 mmol/L^a^	FBG 5.6−6.0 mmol/L	FBG 6.1−6.9 mmol/L	FBG ≥ 7.0mmol/L	*p*Value	FBG < 6.1 mmol/L	FBG 6.1−6.9 mmol/L	FBG ≥ 7.0 mmol/L	*p*Value
*N*(%)	1107 (52.6)	446 (21.2)	253 (12.0)	300 (14.2)		91 (20.7)	58 (13.2)	290 (66.1)	
Gender									
Male, *n* (%)	455 (41.1)	210 (47.1)	123 (48.6)	176 (58.7)	<0.001	39 (42.9）	34 (58.6)	168 (57.9)	0.035
Female, *n* (%)	652 (58.9)	236 (52.9)	130 (51.4)	124 (41.3)		52 (57.1)	24 (41.4)	122 (42.1)	
Age									
Median (IQR)	54 (38−65)	61 (50−68)	62 (51−68.5)	63 (54−70)	<0.001	66 (58−71)	67 (58−72)	65 (58−72)	0.331
>65, *n* (%)	275 (24.8)	151 (33.9)	92 (36.4)	120 (40)	<0.001	49 (53.8)	34 (58.6)	141 (48.6)	0.317
≤65, *n* (%)	832 (75.2)	295 (66.1)	161 (63.6)	180 (60)		42 (46.2)	24 (41.4)	149 (51.4)	
Severity									
Mild, *n* (%)	947 (85.5)	345 (77.4)	161 (63.6)	133 (44.3)	<0.001	65 (71.4)	42 (72.4)	192 (66.2)	0.054
Severe, *n* (%)	126 (11.4)	66 (14.8)	50 (19.8)	56 (18.7)		19 (20.9)	11 (19)	44 (15.2)	
Critical, *n* (%)	34 (3.1)	35 (7.8)	42 (16.6)	111 (37)		7 (7.7)	5 (8.6)	54 (18.6)	
Comorbidities									
Hypertension *n* (%)	237 (21.4)	117 (26.2)	88 (34.8)	101 (33.7)	<0.001	49 (53.8)	26 (44.8)	152 (52.4)	0.516
Hyperlipidemia, *n* (%)	199 (18.0)	91 (20.4)	80 (31.6)	75 (25)	<0.001	16 (17.6)	13 (22.4)	82 (28.3)	0.106
Hyperuricemia, *n* (%)	84 (7.6)	32 (7.2)	21 (8.3)	29 (9.7)	0.612	8 (8.8)	2 (3.4)	19 (6.6)	0.413
Coronary heart disease, *n* (%)	62 (5.6)	28 (6.3)	18 (7.1)	22 (7.3)	0.633	15 (16.5)	11 (19.0)	51 (17.6)	0.927
Stroke, *n* (%)	15 (1.4)	9 (2.0)	12 (4.7)	11 (3.7)	0.003	4 (4.4)	3 (5.2)	10 (3.4)	0.799
Chronic obstructive pulmonary disease, *n* (%)	42 (3.8)	25 (5.6)	9 (3.6)	15 (5)	0.36	3 (3.3)	5 (8.6)	16 (5.5)	0.383
Tumor, *n* (%)	66 (6.0)	18 (4.0)	17 (6.7)	27 (9.0)	0.047	8 (8.8)	0	16 (5.5)	0.017
Clinical symptoms									
Fever, *n* (%)	798 (72.1)	362 (81.2)	207 (81.8)	240 (80.0)	<0.001	59 (64.8)	45 (77.6)	206 (71)	0.241
Muscular soreness, *n* (%)	223 (20.1)	94 (21.1)	53 (20.9)	63 (21.0)	0.969	16 (17.6)	10 (17.2)	51 (17.6)	0.998
Fatigue, *n* (%)	529 (47.8)	252 (56.5)	154 (60.9)	198 (66.0)	<0.001	46 (50.5)	29 (50.0)	175 (60.3)	0.134
Sore throat, *n* (%)	157 (14.2)	51 (11.4)	22 (8.7)	24 (8.0)	0.007	10 (11.0)	6 (10.3)	31 (10.7)	0.992
Dry cough, *n* (%)	581 (52.5)	230 (51.6)	130 (51.4)	133 (44.3)	0.094	50 (54.9)	34 (58.6)	137 (47.2)	0.176
Expectoration, *n* (%)	258 (23.3)	132 (29.6)	72 (28.5)	114 (38.0)	<0.001	21 (23.1)	14 (24.1)	94 (32.4)	0.15
Dyspnea, *n* (%)	168 (15.2)	91 (20.4)	73 (28.9)	120 (40.0)	<0.001	17 (18.7)	16 (27.6)	70 (24.1)	0.41
Consciousness disorder, *n* (%)	31 (2.8)	15 (3.4)	19 (7.5)	53 (17.7)	<0.001	4 (4.4)	2 (3.4)	22 (7.6)	0.312
Complications									
Hypoproteinemia, *n* (%)	172 (15.5)	106 (23.8)	87 (34.4)	139 (46.3)	<0.001	19 (20.9)	17 (29.3)	98 (33.8)	0.064
Respiratory failure, *n* (%)	30 (2.7)	26 (5.8)	27 (10.7)	68 (22.7)	<0.001	5 (5.5)	3 (5.2)	35 (12.1)	0.082
ARDS, *n* (%)	3 (0.3)	5 (1.1)	10 (4.0)	26 (8.7)	<0.001	1 (1.1)	1 (1.7)	9 (3.1)	0.475
Acute kidney injury, *n* (%)	42 (3.8)	21 (4.7)	28 (11.1)	53 (17.7)	<0.001	12 (13.2)	5 (8.6)	45 (15.5)	0.372
Acute hepatic injury, *n* (%)	242 (21.9)	132 (29.6)	93 (36.8)	119 (39.7)	<0.001	22 (24.2)	15 (25.9)	83 (28.6)	0.683
Deep venous thrombosis, *n* (%)	8 (0.7)	15 (3.4)	10 (4.0)	21 (7.0)	<0.001	6 (6.6)	1 (1.7)	11 (3.8)	0.309
Treatment									
Intravenous antibiotics, *n* (%)	391 (35.3)	173 (38.8)	130 (51.4)	205 (68.3)	<0.001	34 (37.4)	31 (53.4)	132 (45.5)	0.146
Arbidol, *n* (%)	801 (72.4)	314 (70.4)	175 (69.2)	188 (62.7)	0.013	70 (76.9)	37 (63.8)	206 (71.0)	0.222
Glucocorticoids, *n* (%)	127 (11.5)	102 (22.9)	78 (30.8)	147 (49.0)	<0.001	20 (22.0)	11 (19.0)	73 (25.2)	0.544
Vasoactive drugs, *n* (%)	93 (8.4)	66 (14.8)	60 (23.7)	115 (38.3)	<0.001	19 (20.9)	10 (17.2)	76 (26.2)	0.257
Chinese medicine, *n* (%)	833 (75.2)	355 (79.6)	189 (74.7)	177 (59)	<0.001	71 (78.0)	45 (77.6)	209 (72.1)	0.424
Nasal catheter or mask oxygen inhalation, *n* (%)	584 (52.8)	282 (63.2)	136 (53.8)	143 (47.7)	<0.001	55 (60.4)	37 (63.8)	160 (55.2)	0.107
Noninvasive ventilator, *n* (%)	18 (1.6)	22 (4.9)	15 (5.9)	51 (17.0)		3 (3.3)	2 (3.4)	32 (11.0)	
Invasive ventilator, *n* (%)	13 (1.2)	15 (3.4)	18 (7.1)	35 (11.7)		3 (3.3)	2 (3.4)	20 (6.9)	
Extracorporeal membrane oxygenation (ECMO), *n* (%)	0	0	0	6 (2.0)		1 (1.1)	0	1 (0.3)	

Abbreviations: ARDS, acute respiratory distress syndrome; COVID‐19, coronavirus diseases 2019; FBG, fasting blood glucose; IQR, interquatile range.

^a^ There were 17 (0.8%) cases with FBG less than 3.9 mmol/L at admission.

### 3.2 The longitudinal trajectory of FBG

**Figure 2:**
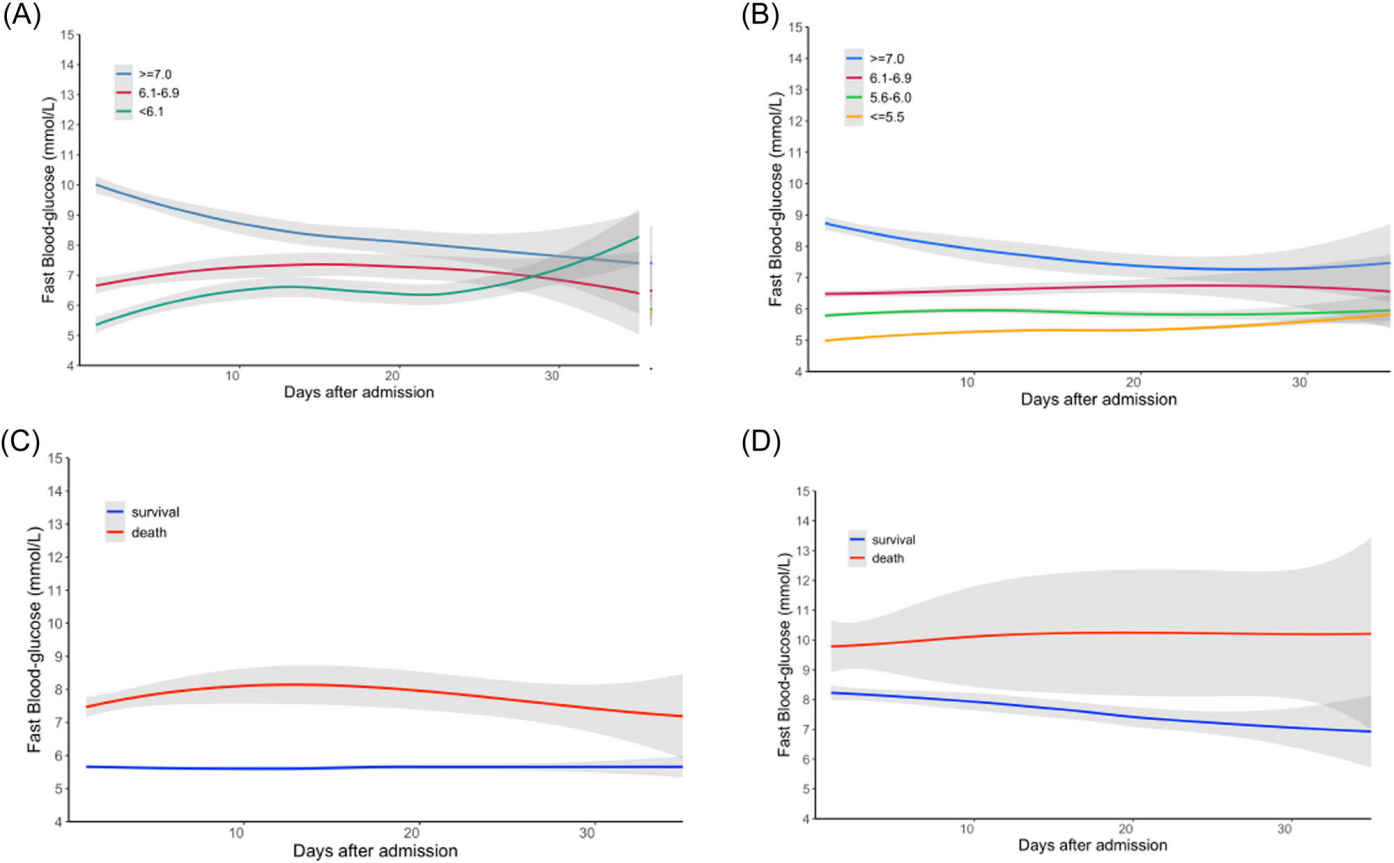
The dynamic changes of fasting blood glucose (FBG) in patients with different admission FBG levels. (A) The Lowess curve of FBG in coronavirus 2019 (COVID‐19) patients without a history of diabetes. (B) The Lowess curve of FBG in COVID‐19 patients with a history of diabetes. (C) The Lowess curve of FBG in the survival and death cases among COVID‐19 patients without a history of diabetes. (D) The Lowess curve of FBG in the survival and death cases among COVID‐19 patients with a history of diabetes

We further collected data of FBG level after admission. The locally weighted scatterplot smoothing (Lowess) method was used to demonstrate the longitudinal trajectory of FBG in nondiabetic COVID‐19 patients with different admission FBG levels (≤5.5, 5.6−6.0, 6.1−6.9, and ≥7.0 mmol/L). In general, the relative position of the longitudinal trajectory of FBG between the four groups remained stable. The FBG level in the ≤5.5 mmol/L group showed an increasing trend and finally stabilized at 5.29 mmol/L (95% confidence interval [CI]: 5.20−5.39). The FBG baseline in the 5.6−6.0 mmol/L group was stable at 5.74 mmol/L (95% CI: 5.6−5.87), controlled at 6.66 mmol/L (95% CI: 6.0−7.32) in the 6.1−6.9 group, and showed a gradual decrease to 7.08 mmol/L (95% CI: 6.44−7.72) within 35 days after admission in the ≥7.0 mmol/L group (Figure 2A). In the COVID‐19 patients with a diabetic history, the FBG level in the ≥7.0 mmol/L group showed a decreasing trend within 35 days after admission and controlled at 8.34 mmol/L (95% CI: 7.69−9.00) and showed an increasing trend in the 6.1−6.9 mmol/L group. The FBG level in the <6.1 mmol/L group stabilized at 6.27 mmol/L (95% CI: 5.71−6.83) in the first 3 weeks, showed a rising trend after 3 weeks, and increased to 8.40 mmol/L (95% CI: 6.90−9.91) finally (Figure 2B).

We further compared the dynamic changes of FBG between the survival and death cases in COVID‐19 patients without a diabetic history. The level of FBG remained stable within the normal range with very little fluctuation in the survival cases. However, it was relatively higher all along with a large fluctuation in the death cases (*p* < 0.001). The FBG baseline gradually increased after 15 days, increased to 8.15 mmol/L (95% CI: 7.57−8.69), and gradually decline to controlled at 7.55 mmol/L (95% CI: 6.46−8.65) in the death cases (Figure 2C). Among the diabetic history group, the baseline level of FBG in the dead cases was similar to that among the nondiabetic history group. The level of FBG in the death cases changed significantly over time with a steadily increasing tendency. However, it continuously declined and was controlled at 7.44 mmol/L (95% CI: 7.04−7.84) in the survival cases (Figure 2D).

### 3.3 One‐year mortality

**Figure 3:**
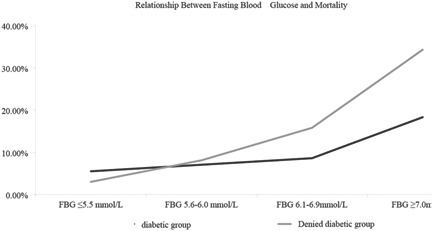
J‐curve associations between fasting blood glucose (FBG) and in‐hospital mortality in the diabetic history group and the nondiabetic history group

We further investigated the relationship between admission FBG level and 1‐year mortality in the COVID‐19 patients. The results showed that, among the 2106 hospitalized COVID‐19 patients without a diabetic history, the 1‐year mortality in the groups of ≤5.5, 5.6−6.0, 6.1−6.9, and ≥7.0 mmol/L were 3.0% (33/1107), 8.1% (36/446), 15.8% (40/253), and 34.3% (103/300), respectively (*p* < 0.001) (Table 2). With the increase of FBG level, 1‐year all‐cause mortality (*p* < 0.001), in‐hospital mortality (*p* < 0.001), and 12‐month postdischarge mortality (*p* = 0.001) gradually increased. Among the diabetic COVID‐19 patients, the 1‐year mortality in the ≥7.0 mmol/L group was significantly higher than those in the other groups (18.3% in the ≥7.0 mmol/L group, 8.6% in the 6.1−6.9 mmol/L group, and 5.5% in the <6.1 mmol/L group, *p* = 0.004). However, there were no significant differences in 12‐month postdischarge mortality among the three groups (*p* = 0.189). After stratification according to different levels of admission FBG, the all‐cause mortality showed a gradually increasing trend and a J‐shaped curve distribution (Figure 3).

**Table 2:** Clinical outcome and lung images of 2545 hospitalized COVID‐19 patients

	Denied diabetes					Diabetes			
	FBG ≤ 5.5 mmol/L^a^	FBG 5.6−6.0 mmol/L	FBG 6.1−6.9 mmol/L	FBG ≥ 7.0 mmol/L	*p*Value	FBG < 6.1 mmol/L	FBG 6.1−6.9 mmol/L	FBG ≥ 7.0 mmol/L	*p*Value
*N*(%)	1107 (52.6)	446 (21.2)	253 (12.0)	300 (14.2)		91 (20.7)	58 (13.2)	290 (66.1)	
1‐year all‐cause mortality, *n* (%)	33 (3.0)	36 (8.1)	40 (15.8)	103 (34.3)	<0.001	5 (5.5)	5 (8.6)	53 (18.3)	0.004
In‐hospital mortality, *n* (%)	24 (2.2)	33 (7.4)	36 (14.2)	95 (31.7)	<0.001	4 (4.4)	5 (8.6)	46 (15.9)	0.01
1‐year postdischarge mortality, *n* (%)	9 (0.8)	3 (0.7)	4 (1.8)	8 (3.9)	0.013	1 (1.1)	0	7 (2.9)	0.189
Follow‐up time									
After admission, median (IQR)	13.0 (12.9−13.3)	13.1 (13.0−13.3)	13.2 (13.0−13.3)	13.2 (13.0−13.4)	<0.001	11.1 (11.0−11.4)	11.1 (10.9−11.4)	11.1 (10.7−11.4)	0.217
After discharge, median (IQR)	12.3 (12.1−12.7)	12.2 (12.1−12.6)	12.2 (11.9−12.6)	12.2 (11.9−12.6)	0.072	10.2 (9.9−10.5)	10.2 (9.9−10.8)	10.2 (10.0−10.6)	0.37

Abbreviations: COVID‐19, coronavirus diseases 2019, FBG, fasting blood glucose; IQR, interquatile range.

^a^ Among 17 patients with FBG < 3.9 mmol/L at admission, 1‐year all‐cause mortality was 3 (17.6%), in‐hospital mortality was 3 (17.6%), and 12‐month postdischarge mortality was 0 (0%).

### 3.4 Clinical sequelae at 1 year after discharge

Because COVID‐19 patients had multiple complications during hospitalization, we focused on the clinical sequelae and lung images after discharge (Table 3). We asked patients about their symptoms through telephone and outpatient follow‐up. For patients who denied a history of diabetes, patients in the ≥7.0 mmol/L group had a higher risk of chest tightness, (*p* = 0.025), but other sequelae such as fatigue, cough, shortness of breath, and low back pain showed no significant difference. For lung images, patients in the ≥7.0 mmol/L group were more likely to have slower recovery of abnormal lung scans (*p* < 0.001) (Table 3). However, among COVID‐19 patients with diabetic history, there were no significant differences in clinical sequelae and lung images between patients with different admission FBG levels.

**Table 3:** Sequelae of 2545 hospitalized nondiabetic COVID‐19 patients

	Denied diabetes					Diabetes			
	FBG ≤ 5.5 mmol/L^a^	FBG 5.6−6.0 mmol/L	FBG 6.1−6.9 mmol/L	FBG ≥ 7.0 mmol/L	*p*Value	FBG 5.6−6.0 mmol/L	FBG 6.1−6.9 mmol/L	FBG ≥ 7.0 mmol/L	*p*Value
Sequelae	202 (18.8)	81 (19.8)	41 (19.2)	46 (23.4)	0.53	15 (17.4)	13 (24.5)	54 (22.8)	0.516
Total cases, *n* (%)	54 (5.0)	15 (3.7)	14 (6.6)	6 (3.0)	0.248	6 (7.0)	5 (9.4)	13 (5.5)	0.574
Fatigue, *n* (%)	46 (4.3)	18 (4.4)	9 (4.2)	12 (6.1)	0.722	2 (2.3)	5 (9.4)	13 (5.5)	0.183
Chest tightness, *n* (%)	49 (4.6)	33 (8.0)	10 (4.7)	16 (8.1)	0.025	3 (3.5)	3 (5.7)	14 (5.9)	0.665
Cough, *n* (%)	40 (3.7)	7 (1.7)	10 (4.7)	10 (5.1)	0.096	3 (3.5)	5 (9.4)	5 (2.1)	0.067
Shortness of breath, *n* (%)	39 (3.6)	25 (6.1)	6 (2.8)	10 (5.1)	0.117	5 (5.8)	1 (1.9)	17 (7.2)	0.262
Lung images									
Normal, *n* (%)	736 (68.5)	235 (57.3)	125 (58.7)	111 (56.3)	<0.001	44 (51.2)	23 (43.4)	130 (54.9)	0.309
Focal ground‐glass opacity, *n* (%)	205 (19.1)	106 (25.9)	57 (26.8)	62 (31.5)	<0.001	24 (27.9)	21 (39.6)	75 (31.6)	0.351
Fibrosis, *n* (%)	40 (3.7)	18 (4.4)	13 (6.1)	5 (2.5)	0.271	3 (3.5)	3 (5.7)	7 (3.0)	0.657

Abbreviations: COVID‐19, coronavirus diseases 2019; FBG, fasting blood glucose.

^a^ Among 17 patients with FBG < 3.9 mmol/L at admission.

### 3.5 Multivariate Cox risk regression analysis

**Figure 4:**
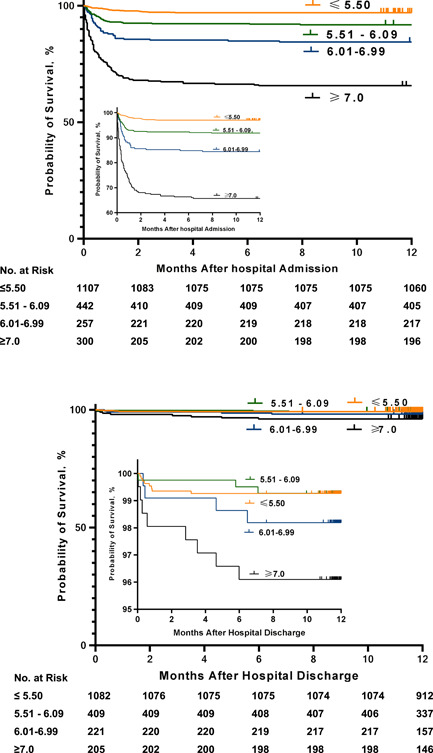
Kaplan−Meier survival curves of 2106 coronavirus 2019 patients without a diabetic history in different admission fasting blood glucose levels. (A) Kaplan−Meier survival curve of 1‐year all‐cause mortality after admission. (B) Kaplan−Meier survival curve of 12‐month postdischarge mortality in hospitalized survivors

We used Cox regression analysis and Kaplan−Meier survival curve to explore the risk of death between the history of diabetes and multiple factors in COVID‐19 patients. In patients who denied diabetes history, the risk of death was positively associated with the level of admission FBG. With the increase of FBG, the all‐cause mortality increased (*p* < 0.01). Multivariate analysis showed that age (>65 years, hazard ratio [HR] = 3.05, 95% CI: 2.26−4.12), gender (male, HR = 1.94, 95% CI: 1.44−2.6), tumor (HR = 2.36, 95% CI: 1.65−3.38), stroke (HR = 2.81, 95% CI: 1.76−4.51), hyperuricemia (HR = 2.07, 95% CI: 1.37−3.13), chronic kidney disease (HR = 2.16, 95% CI: 1.04−4.52), FBG 5.6−6.0 mmol/L (HR = 2.67, 95% CI: 1.66−4.28), 6.1−6.9 mmol/L (HR = 4.78, 95% CI: 3.01−7.61), and FBG ≥ 7.0 mmol/L (HR = 10.63, 95% CI: 7.15−15.83) were independent risk factors for 1‐year all‐cause mortality (Table 4). In patients who with a diabetic history. Multivariate analysis showed that age (>65 years, HR = 2.5, 95% CI: 1.46−4.29), hyperuricemia (HR = 2.76, 95% CI: 1.3−5.86), and FBG ≥ 7.0 mmol/L (HR = 3.9, 95% CI: 1.56−9.77) were independent risk factors for 12‐month postdischarge mortality (Table 5); (Figure 4).

**Table 4:** Multivariate prediction of 1 year mortality in 2106 COVID‐19 patients without a diabetic history

Variables	Odds ratio	95% CI	*p*
FBG ≤ 5.5 mmol/L	1 (ref)		<0.001
FBG 5.6−6.0 mmol/L	2.67	1.66−4.28	<0.001
FBG 6.1−6.9 mmol/L	4.78	3.01−7.61	<0.001
FBG ≥ 7.0 mmol/L	10.63	7.15−15.83	<0.001
Male	1.94	1.44−2.6	<0.001
Age > 65	3.05	2.26−4.12	<0.001
Tumor	2.36	1.65−3.38	<0.001
Coronary heart disease	1.2	0.77−1.88	0.428
Stroke	2.81	1.76−4.51	<0.001
Hypertension	0.9	0.67−1.21	0.474
Hyperuricemia	2.07	1.37−3.13	0.001
Chronic obstructive pulmonary disease	0.95	0.56−1.60	0.833
Chronic kidney disease	2.16	1.04−4.52	0.04

Abbreviations: CI, confidence interval; COVID‐19, coronavirus diseases 2019; FBG, fasting blood glucose.

**Table 5:** Multivariate prediction of 1‐year mortality in 439 COVID‐19 patients with a diabetic history

Odds ratio	Odds ratio	95% CI	*p*
FBG < 6.1 mmol/L	1(ref)		0.005
FBG 6.1−6.9 mmol/L	1.71	0.49−5.91	0.399
FBG ≥ 7.0 mmol/L	3.9	1.56−9.77	0.004
Age > 65	2.5	1.46−4.29	0.001
Hyperuricemia	2.76	1.3−5.86	0.008

Abbreviations: CI, confidence interval; COVID‐19, coronavirus diseases 2019; FBG, fasting blood glucose.

## 4 DISCUSSION

In this bidirectional cohort study, our study shows that the 1‐year all‐cause mortality rate and in‐hospital mortality rate of COVID‐19 patients were J‐curve correlated with FBG. FBG ≥ 7.0 mmol/L can be a predictive factor of 1‐year all‐cause mortality in COVID‐19 patients, independent of diabetes history. FBG ≥ 7.0 mmol/L has an advantage in predicting the severity, clinical sequelae, and pulmonary absorption in COVID‐19 patients without a history of diabetes. But the results found that fasting glucose at admission was not significantly superior in predicting disease course and prognosis in patients with a history of diabetes. Previous studies have shown that FBG may be associated with a poor prognosis in COVID‐19 patients and early intervention can help improve the prognosis.^2^, ^5^, ^6^, ^11^ Lazarus et al.^7^ proved an explosion‐response gradient relationship between admission FBG level and severity degree in COVID‐19 patients, Wang et al. demonstrated that FBG was an independent predictor of poor prognosis at 28 days in COVID‐19 patients, showing no association with the history of diabetes.^11^ Diabetes is associated with the severity and mortality of COVID‐19. However, it was not possible to prove whether diabetes was an independent factor or whether it was just a confounding factor. Many diseases, such as old age, hypertension, cardiovascular disease, and obesity, often coexist with diabetes, and each of these comorbidities has been shown to be associated with the severity and mortality of COVID‐19. Therefore, the role of fasting glucose at admission in predicting the disease course and prognosis of COVID‐19 patients with a history of diabetes is likely to be superimposed by other factors and not significantly superior. COVID‐19 brought about not only clinical manifestations in the acute phase, but also many complications afterward, and the pathologic changes in the lung caused by COVID‐19 will take a long time to restore. Therefore, in addition to the survival of the patients, we also focused on the related complications and pulmonary changes of FBG at 1 year after discharge. We found that, among COVID‐19 patients without a diabetic history, those whose admission ≥7.0 mmol/L were more likely to have chest tightness and slower recovery of abnormal lung images at 1 year after discharge. Roncon et al. reported that COVID‐19 patients with hyperglycemia or diabetes were more likely to undergo ICU care.^12^ Lazarus et al. reported that hyperglycemia promotes the progression of COVID‐19.^3^, ^4^, ^13^ Diabetic microvascular lesions in the respiratory tract may impair alveolar gas exchange and lung compliance, resulting in impaired lung function and reduced absorption of lung inflammation.^14^ Caruso et al. confirmed that diabetes and hyperglycemia could cause pulmonary remodeling and respiratory restriction.^15^ We found that the levels of admission FBG in COVID‐19 patients had a significant impact on the incidence of clinical complications, the use of glucocorticoids, vasoactive drugs, and mechanical ventilation. It suggested that, with the increasing of admission FBG level, patients without a diabetic history tended to be more severe, need more intensive treatment, and have a worse prognosis. Therefore, we believe that the level of admission FBG, a risk predictor for COVID‐19, needs to be further stratified so as to predict the mortality and the incidence of long‐term complications.

Patients with FBG > 6.1 mmol/L accounted for 26.2% (553/2106) and a total of 14.2% (300/2106) patients had blood glucose ≥7.0 mmol/L. These results indicated that our study included both undiagnosed diabetic patients and nondiabetic patients with hyperglycemia caused by an acute blood‐glucose disorder. Similar to a previous study, COVID‐19 patients might suffer from stress hyperglycemia.^16^ Therefore, the relationship between the severity of COVID‐19 and hyperglycemia may be an interaction. On the one hand, the infection of SARS‐CoV‐2 can cause impaired glucose metabolism. SARS‐CoV‐2 enters the host via angiotensin‐converting enzyme 2 (ACE2), which is also expressed in pancreatic beta cells, thus providing a pathway for the virus to enter and destroy the islets and resulting in defective insulin production and hyperglycemia in COVID‐19 patients^12^, ^17^, ^18^ SARS CoV‐2 infection leads to increased release of inflammatory factors, which causes insulin resistance and thus contributes to elevated blood glucose.^7^, ^17^, ^18^ Hyperglycemia inhibits the chemotaxis of neutrophils, reduces the phagocytosis of neutrophils, macrophages and monocytes, and impairs innate cell‐mediated immunity.^19^ In COVID‐19 patients, the proportion of proinflammatory Th17 CD4^+^ T cells and cytokines is elevated, while peripheral CD4^+^and CD8^+^ T cells are decreased. Therefore, hyperglycemia may induce impaired antiviral interferon response and delayed activation of Th1/Th17, contributing to a high inflammatory response.^20^ On the other hand, a large part of these patients developed acute kidney injury and abnormal liver function. As liver and kidney are key organs for glucose metabolism, the level of blood glucose would be significantly impacted.^2^ These patients were more likely to be treated with glucocorticoids, so the blood glucose might show a large fluctuation. According to the dynamic analysis of FBG in COVID‐19 patients without a diabetic history, we found that the relative position of the longitudinal trajectory remained stable at the beginning in different admission FBG groups. Interestingly, in diabetic COVID‐19 patients with admission FBG < 6.1 mmol/L, we observed that the level of FBG tended to be stable in the first 3 weeks and showed an increasing trend after 3 weeks. This might be related to the disease aggravation and the enhanced release of proinflammatory cytokines caused by infection, thus leading to insulin resistance.^11^, ^21^ Hyperglycemia induces oxidative stress and leads to endothelial dysfunction,^7^, ^19^ which may lead to further pulmonary microangiopathy. These studies might explain the strong association of elevated FBG levels with clinical complications, disease severity, and mortality among COVID‐19 patients without a diabetic history and supported the direct association between FBG levels and the progression of COVID‐19.

This study has several limitations. First, we did not cover glycated hemoglobin (HbA1c), a long‐term glycemic control indicator that helps distinguish patients with poor long‐term glycemic control from those with stress hyperglycemia. Moreover, most of the COVID‐19 patients were from the designated hospitals for critical patients during the pandemic, which might cause a bias of higher mortality rates. Hence, we tried to enlarge the sample size to reduce the bias.

In conclusion, our study shows that FBG ≥ 7.0 mmol/L can be a predictive factor of 1‐year all‐cause mortality in COVID‐19 patients, independent of diabetes history. FBG ≥ 7.0 mmol/L has an advantage in predicting the severity, clinical sequelae, and pulmonary absorption in COVID‐19 patients without a history of diabetes. Early detection, timely treatment, and strict control of blood glucose when finding hyperglycemia in COVID‐19 patients (with or without diabetes) are critical for their prognosis.

## AUTHOR CONTRIBUTIONS

Zehai Tang and Chen Chai conceived the project. Zehai Tang, Shoupeng Li, Kui Chen, Hongxiang Wang, Wendan Wang, Dunshuang Wei, Cao Peng, and Qi Sun collected analyzed the data. Zehai Tang and Chen Chai wrote the manuscript.

## CONFLICTS OF INTEREST

The authors declare no conflicts of interest.

## ETHICS STATEMENT

This study was approved by the Institutional Ethics Committee of Union Hospital (No. 2021‐0005‐01) and the Institutional Ethics Committee of the Central Hospital of Wuhan (No. 2020‐7). The data used were deidentified. No reference has been made at any point to individually identifiable data. All of the data used in this study come from Wuhan Union Hospital and its affiliated hospitals or the Central Hospital of Wuhan.

## Supporting information

Supporting information.

## Data Availability

The data that support the findings of this study are available from the corresponding author upon reasonable request.
