# Supplementary material for: Effect of elevated fasting blood glucose level on the 1‐year mortality and sequelae in hospitalized COVID‐19 patients: A bidirectional cohort study
Source: J Med Virol. 2022 Apr 19;94(7):3240–50. doi: 10.1002/jmv.27737 (PMC9088618; doi:10.1002/jmv.27737)
Supplement: Supplementary file 1 — Supporting information. [file JMV-94-3240-s001.doc]

**Effect of elevated fasting blood glucose level on the one-year mortality and sequelae in hospitalized COVID-19 patients: a bidirectional cohort study**

**Supplementary Data**

[Supplementary Table . Definitions of COVID-19, Variables and Outcomes 2](#__RefHeading___Toc85986497)

[Supplementary Case Report Form 5](#__RefHeading___Toc85986498)

[Confirmation of eligibility 5](#__RefHeading___Toc85986499)

[**Institutional Data** 6](#__RefHeading___Toc85986500)

[Demographics, Symptoms, Severity, Comorbidities, Home Medications 7](#__RefHeading___Toc85986501)

[Vital Signs and Severity-of-Illness in the First Days following Admission in Hospital 13](#__RefHeading___Toc85986502)

[Labs and Physiologic Parameters in the First Days following Admission in Hospital 15](#__RefHeading___Toc85986503)

[Medications and adjuvant therapy 19](#__RefHeading___Toc85986504)

[Mortality, Length of Stay 23](#__RefHeading___Toc85986505)

[Supplementary Follow-Up Studies 25](#__RefHeading___Toc85986506)

[Follow-up Questionnaire 25](#__RefHeading___Toc85986507)

This supplementary material has been provided by the authors to give readers additional information about their work.

## Supplementary Table . Definitions of COVID-19, Variables and Outcomes

| **COVID-19 Severity** | |
| --- | --- |
| Asymptomatic | Laboratory-confirmed positive COVID-19 test, none of the typical symptoms |
| Mild | Being a bit more breathless than normal on exercise, but they are not out of breath on normal household activity |
| Moderate | More breathless and tend to have an increased heart rate, particularly if they are moving around |
| Severe | It conforms to any of the following: 1) shortness of breath during resting conditions, RR ≥ 30 times per minute; 2) SPO2 ≤ 93% when breathe in the air during resting conditions; 3) PaO2/FiO2 ≤ 300mmHg; 4) The clinical symptoms progressed and pulmonary imaging showed that the lesion had a significant progression of more than 50% within 24 - 48 hours |
| Critical | Respiratory failure requires mechanical ventilation, go into shock Combined with other organ failure requires intensive care in ICU |
| **Coexisting Conditions** | |
| Smoking | Does not include vaping or smoking of non-tobacco products.   1. Non- smoker 2. Former smoker 3. Current smoker |
| Hypertension | The systolic blood pressure is higher than 140mmHg or diastolic blood pressure is higher than 90mmHg |
| Diabetes mellitus | Insulin versus non-insulin dependent |
| Chronic obstructive pulmonary disease | Persistent respiratory symptoms and airflow limitation |
| Coronary artery disease | Any history of angina, myocardial infarction, or coronary artery bypass graft surgery |
| Congestive heart failure | Heart failure with preserved versus reduced ejection fraction |
| Chronic kidney disease | Renal impairment at baseline (estimated glomerular filtration rate [eGFR] <60 mL/min/1.73 m ^2^) on at least two consecutive values at least 12 weeks apart prior to hospital admission. |
| End-stage kidney disease | On hemodialysis or peritoneal dialysis |
| Cerebrovascular disease | Includes stroke, carotid stenosis, vertebral stenosis and intracranial stenosis. Restrictions in blood flow may occur from vessel narrowing (stenosis), clot formation (thrombosis), blockage (embolism) or blood vessel rupture (hemorrhage). |
| **Complication** | |
| Acute kidney injury | Doubling of serum creatinine from baseline or need for renal replacement therapy (RRT), the hospital admission value was used as the baseline. |
| Acute liver injury | Bilirubin >3.0 mg/dl *and* either AST>100 units per liter or ALT>100 units per liter |
| Acute cardiac injury | Troponin T or I > the 99th percentile upper reference limit of normal for that lab |
| Acute respiratory distress syndrome | PaO2 / FiO2 ≤ 300mmHg，PEEP or CPAP ≥ 5cmH2O  and mechanically ventilated |
| Congestive heart failure (new onset) | Includes both heart failure with preserved and reduced ejection fraction |
| Disseminated intravascular Coagulation | Systemic activation of blood coagulation, which generates intravascular thrombin and fibrin, resulting in the thrombosis of small- to medium-sized vessels and ultimately organ dysfunction and severe bleeding |
| Gastrointestinal bleeding | Includes non-venous upper gastrointestinal bleeding or lower gastrointestinal bleeding |
| Respiratory failure | Requirement for invasive mechanical ventilation |
| Secondary Infection | Suspected or confirmed new infection other than COVID-19 that developed after admission in hospital. Pneumonia (including ventilator-associated), urosepsis, biliary sepsis, bacteremia, etc. |
| Shock | Requirement for 2 or more vasopressors |
| Thromboembolic event | Deep venous thrombosis, pulmonary embolism, stroke, etc. |
| **Medication Treatments** | |
| Antiviral drug | Include Arbidol hydrochloride, Ribavirin, interferon, Oseltamivir, Ganciclovir, entecavir, peramivir, etc. |
| Intravenous antibiotics | Include vancomycin, piperacillin-tazobactam, azithromycin, cephalosporins, quinolones, etc. |
| Intravenous antifungal | Include Voriconazole, amphotericin B，caspofungin, etc. |
| Anticoagulation | Include Heparin, enoxaparin (Lovenox), Bivalirudin (Angiomax), Argatroban, etc. |
| Intravenous corticosteroids | Include Decadron, methylprednisolone，hydrocortisone, etc. |
| Granulocyte colony-stimulating factor | Recombinant human granulocyte colony-stimulating factor |
| Chinese medicine | Include Qingfei Paidu Decoction, HuiFu Presciption, etc. |
| **Organ Support Therapy** | |
| Oxygen therapy | Include Low-flow oxygen therapy, High-flow oxygen therapy (HFT) |
| mechanical ventilation | Include Non-invasive ventilation (NIV), Invasive mechanical ventilation (IMV) |
| Extracorporeal membrane oxygenation (ECMO) | Vena-venous, vena-arterial, or vena-arterial-venous |
| Renal replacement therapy | CRRT, intermittent hemodialysis, peritoneal dialysis, other |
| Cause of Death | ARDS/respiratory failure, congestive heart failure, septic shock, kidney failure, liver failure, etc. |
| Abbreviations: ALT, alanine transaminase; AST, aspartate transaminase; COVID-19, coronavirus-19; CRRT, continuous renal replacement therapy; RR, respiratory rate; FiO2, fraction of inspired oxygen; PaO2, partial pressure of oxygen; PEEP, positive end-expiratory pressure ; CPAP, continuous positive airway pressure; ICU, intensive care unit; DVT, deep vein thrombosis; eGFR, estimated glomerular filtration rate; PE, pulmonary embolism; COPD, chronic obstructive pulmonary disease; CHD, coronary Heart Disease | |

**Data Collection and Validation**

Data were collected using electronic case of report form, all data are uploaded to MySQL, a secure application. Wherever possible, data were captured using checkboxes rather manual entry to minimize keystroke errors. For data that required keystroke entry (e.g., laboratory values), we implemented validation ranges to flag potential errors in real-time. We also implemented automated data validation rules to flag errors in dates. Finally, all data were verified by study personnel at each site by manual review of electronic medical records, and values that appeared incongruent or out of range were manually validated by confirming the accuracy of the data with the collaborator who entered it.

**Analysis of 1-year all-cause mortality among hospitalized patients**

All patients enrolled in the study who were discharged alive from the hospital were followed-up，after manually reviewed their case, we called them or in the outpatient to certain their 1-year survival status. Each patient had at least one follow-up result.

**Multivariable Modeling of Patients Clinic Date and 1-year Mortality**

We performed multivariable regression modeling to estimate the association between several variables and the primary outcome. We prespecified the following variables for inclusion in the multivariable model based on clinical knowledge, biologic plausibility, and completeness of data.

## Supplementary Case Report Form

### Confirmation of eligibility

| Study ID |  |
| --- | --- |
| **The questions in this instrument will confirm whether the patient is eligible** | |
| Please read this document carefully before you begin entering data. | |
| Laboratory-confirmed diagnosis of COVID-19? | 〇Yes  〇No |
| STOP, THIS PATIENT IS NOT ELIGIBLE | |
| Date the positive COVID-19 test was obtained  -Refers to the date the patient was tested, not the date the result came back  -If multiple tests were sent, provide the date of the initial positive test, even if it was obtained at an outside facility  -Ok to use an estimate if exact date unavailable |  |
| Hospitalized for illness related to COVID-19?  Do not include patients admitted in hospital for reasons unrelated to COVID-19 who later tested positive on "routine surveillance" only and never showed signs or symptoms consistent with COVID-19. | 〇Yes  〇No |
| STOP, THIS PATIENT IS NOT ELIGIBLE | |
| THIS PATIENT IS ELIGIBLE, PROCEED WITH DATA ENTRY | |
| Additional Notes (if any) | |

**Institutional Data**

| Enter your name:The name of the person entering data, not the name of the patient |  |
| --- | --- |
| Enter your email address |  |
| Which hospital the patient came from  , West, Center | 〇West Campus, Union Hospital  〇Tumor Center, Union Hospital  〇The Main Campus, Union Hospital  〇The Central Hospital of Wuhan  〇Other |
| Additional Notes (if any) |  |

### Demographics, Symptoms, Severity, Comorbidities, Home Medications

| Age（in years） |  | |
| --- | --- | --- |
| Gender | 〇Male  〇Female | |
| Habitual residence |  | |
| Contact information |  | |
| Pregnant at the time of admission? | 〇Yes  〇No | |
| How many weeks pregnant on admission? | (Round up or down to the nearest integer value) | |
| Enter the outcome of the fetus by the end of the hospitalization (e.g., "both the mother and fetus survived the hospitalization", or "fetus terminated at week XXX due to XXX", etc.) |  | |
| Source of admission to the Hospital | 〇Emergency department  〇Outpatient  〇Transfer from another hospital  〇Other | |
| Suspected setting in which COVID-19 infection occurred  *Healthcare worker is defined as a doctor, nurse, technician, or other medical professional who provides direct care to patients (do not include ancillary staff such as clerks, pharmacists, or kitchen/cleaning staff) | 〇Community-acquired  〇Nosocomial  〇Occupational(healthcare worker)*  〇Unknown | |
| Type of healthcare worker | 〇Doctor  〇Nurse  〇Other | |
| Date symptoms first began?  Ok to use an estimate if exact date is unavailable |  | |
| Symptom(s) that occur prior to admission and during hospitalization (select all that apply) | 〇Fever  〇Chills  〇Headache or dizzy  〇Myalgias  〇Fatigue  〇Rhinorrhea  〇Sore throat  〇Dry cough  〇Expectoration  〇Hemoptysis  〇Chest congestion  〇Dyspnea  〇Nausea or vomiting  〇Abdominal pain  〇Diarrhea  〇Consciousness disorder | |
| Other symptom(s)  that occur prior to admission and during hospitalization? Examples include chest pain/tightness, dizziness, and anosmia (lack of smell) |  | |
| Severity of illness at admission  *Non-severe is defined as Laboratory-confirmed positive COVID-19 test，With or without fever, respiratory symptoms, and imaging findings of pneumonia may or may not be visible  **Severe is defined based on Non-severe，In addition, it conforms to any of the following：1.shortness of breath during resting conditions, RR≥30 times per minute,2. SPO2≤93% when breathe in the air during resting conditions 3.PaO2/FiO2≤300mmHg 4. The clinical symptoms progressed and pulmonary imaging showed that the lesion had a significant progression of more than 50% within 24-48 hours 5.Respiratory failure requires mechanical ventilation 6、go into shock 7. Combined with other organ failure requires intensive care in ICU | Non-severe | 〇Asymptomatic  〇Mild  〇Moderate |
|  | Severe | 〇Severe  〇Critical |
| Active malignancy prior to admission  Active malignancy is defined as any malignancy (other than non-melanoma skin cancer) that was treated in the prior year | 〇Yes  〇No  〇Unknown | |
| Subtype of malignancy | 〇Papillary thyroid carcinoma  〇Invasive breast carcinomia  〇Cervical cancer  〇Ovary cancer  〇Endometrial cancer  〇Prostatic cancer  〇Bladder cancer  〇Penile cancer  〇Renal cancer  〇Testicular cancer  〇Gastric cancer  〇Colon cancer  〇Rectal cancer  〇Hepatocellular cancer  〇Pancreas cancer  〇Small cell carcinoma  〇Chondro-osseous tumors  〇Nasopharyngeal cancer  〇Oligodendroglioma  〇Basal cell carcinoma  〇Multiple myeloma  〇Non-Hodgkin lymphoma  〇Chronic lymphoblastic leukemia  〇Acute lymphoblastic leukemia  〇Acute myelogenous leukemia  〇Myelodysplastic syndrome  〇Other | |
| Cardiovascular and pulmonary comorbidities prior to admission  *CAD includes any history of angina, myocardial infarction, or coronary artery bypass graft surgery  **CHF includes both HFrEF and HFpEF | 〇Diabetes mellitus  〇Hypertension  〇Coronary artery disease(CAD)*  〇Congestive heart failure(CHF)**  〇Other heart diseases  〇COPD  〇Other lung disease  〇None of the above | |
| Was the diabetes insulin-dependent or non-insulin-dependent? | 〇Insulin-dependent  〇Non-Insulin-dependent | |
| Enter the other heart disease |  | |
| Enter the other lung disease |  | |
| Tobacco smoking status  Do not include vaping or smoking of non-tobacco products | 〇No smoker  〇Former smoker  〇Current smoker  〇Unknown | |
| How many pack-years of smoking history? If not available enter, "N/A" |  | |
| History of alcohol abuse | 〇Yes  〇No  〇Unknown | |
| Additional comorbidities prior to admission  *Chronic Kidney Disease (CKD) is defined as a baseline eGFR< 60 on at least two consecutive values at least 12 weeks apart prior to hospital admission. If not available, defined as per medical history  **Chronic liver disease includes cirrhosis, autoimmune cirrhosis, primary biliary cirrhosis, and others | 〇CKD*  〇Chronic liver disease**  〇Other immunodeficiency  〇None of the above | |
| Type of chronic liver disease | 〇Alcohol-related cirrhosis  〇Virus-related cirrhosis  〇Autoimmune cirrhosis  〇Primary biliary cirrhosis  〇Other | |
| Enter other chronic liver disease |  | |
| Enter the other immunodeficiency |  | |
| Anti-hypertensive meds prior to hospital admission (select all that apply)  ACE-Is include captopril, ramipril, etc.  ARBs include losartan, valsartan, irbesartan, etc.  MRAs include spironolactone and furosemide, etc. | 〇ACE-I  〇ARB  〇Mineralocorticoid receptor antagonist(MRA)  〇Beta-blocker  〇Other anti-hypertensive  〇None of the above | |
| Enter the other anti-hypertensive(s) If entering more than one, separate with commas. |  | |
| Other meds prior to hospital admission (select all that apply).  Note: only refers to home meds (don't include meds started at an outside hospital)  Statins include atorvastatin, pravastatin, rosuvastatin, and others  NSAIDs include ibuprofen (Advil, Motrin), naproxen (Aleve), and others | 〇Stain  〇NSAID  〇Aspirin  〇None of the above | |
| Therapeutic anticoagulants prior to admission Do not include anti-platelet agents such as aspirin or clopidogrel (Plavix) as "other anticoagulant" | 〇Warfarin(Coumadin)  〇Lovenox (Enoxaparin)  〇Apixaban(Eliquis)  〇Rivaroxaban(Xarelto)  〇Other anticoagulant  〇None of the above | |

### Vital Signs and Severity-of-Illness in the First Days following Admission in Hospital

| Unless otherwise specified, the timing of the data below refers to the first day in the following admission in the hospital, defined as the 24h period from midnight PRIOR to admission to midnight AFTER admission in hospital. For example, if a patient is admission at 10pm, 22 of the 24 hours for first day would actually be from prior to arrival in hospital (e.g., from the ED or other hospital). | |
| --- | --- |
| Date of hospital admission  If hospitalized elsewhere and then transferred to your hospital, enter the date of the initial hospitalization (e.g., if initially at "Hospital A"  and then transferred to "Hospital B", enter the date admitted to Hospital A) |  |
| Weight on first day in admission  -Can enter in either kg or jin  -If none available, enter, "N/A" |  |
| Units for weight  Be sure to select the correct unit | 〇Kilograms  〇Jin |
| Highest temperature on first day in admission |  |
| Units for temperature | 〇Fahrenheit  〇Celsius |
| Lowest systolic blood pressure on first day  Irrespective of whether on pressors or not |  |
| Highest heart rate on first day |  |
| Highest respiratory rate on first day |  |
| What type of oxygen delivery did the patient require on first day?  -Invasive mechanical ventilation refers to mechanical or non-rebreather mask ventilation delivered via endotracheal or tracheal  -If more than one, select the highest level of support | 〇Not requiring supplement oxygen  〇Requiring supplement oxygen  〇High-flow nasal canula for oxygen  therapy (HFNC)  〇Non-invasive positive pressure  〇Ventilation (NIV)  〇Invasive mechanical ventilation  (IMV)  〇Extracorporeal membrane  oxygenation (ECMO) |
| Did the patient have any confirmed or suspected infection(s) on first day following admission other than COVID-19?  *Only select bacterial pneumonia if the patient had positive cultures (e.g., sputum or blood), appositive urine antigen for pneumococcus or legionella, or a new infiltrate on chest imaging (CXR or CT) suspected to be separate from COVID-related pneumonia  **Examples of viral respiratory infections include influenza, parainfluenza, and RSV  ***Only if confirmed by blood cultures and/or cardiac imaging | 〇Bacterial Pneumonia*  〇Viral respiratory infections **  〇Sepsis  〇Cellulitis  〇Bacteremia or endocarditis***  〇Other  〇None of the above |
| Enter the viral respiratory infection |  |
| Enter the other infection |  |
| Enter the number of antibiotics the patient was treated with in the first day following admission -Do not include antivirals, antifungals, or antimalarials  -Commonly used antibiotics include vancomycin, piperacillin-tazobactam, azithromycin, cephalosporins, quinolones, etc. | 〇0  〇1  〇2  〇3  〇4  〇5 or more |
| Enter the number of antivirals the patient was treated with in the first day following admission -Do not include antibiotics, antifungals, or antimalarials  -Commonly used antibiotics Arbidol hydrochloride, Ribavirin, interferon, Oseltamivir, Ganciclovir, entecavir, peramivir, etc. | 〇0  〇1  〇2  〇3  〇4  〇5 or more |

### Labs and Physiologic Parameters in the First Days following Admission in Hospital

| The questions below refer to the first day following admission in hospital. If the patient was in the hospital for less than one day or until discharged from the hospital or death (This patient should be excluded).  Each Day is a discrete 24h period, from midnight to midnight. Day1 refers to the 24h period from the midnight PRIOR to admission to the midnight AFTER admission in hospital.  Select the first lab value of each patient after admission, If there is no value available, leave the field blank (i.e., do NOT write, "N/A" here)  -If a lab is below assay (e.g., Troponin< 6), enter 0  -If a lab is above assay (e.g., IL-6>3,000), enter the upper limit of the assay (e.g., "3,000")  -Do not include any units or "%" in these fields; only enter numbers  -If both an arterial and venous lactate are available on the same day, enter the arterial value, otherwise enter the venous value  Please pay close attention to the units, particularly for CRP, D-dimer, and troponin, and convert to the units we are using if needed. For example, if your lab reports troponin in ng/ml, multiply the values by 1,000 to convert to ng/L | |
| --- | --- |
| Physiologic parameters |  |
| Any Mechanical support following admission?  Check the box if the patient received any of the associated therapies on that day  * For patients who received RRT intermittently (e.g., every other day), continue to check the box as long as they are RRT-dependent | 〇Non-invasive mechanical  ventilation  〇Invasive mechanical ventilation  〇ECMO  〇Renal Replacement Therapy(RRT)*  〇None of the above |
| Date Non-invasive mechanical ventilation support |  |
| Date Non-invasive mechanical ventilation support was removed |  |
| Date invasive mechanical ventilation support |  |
| Date invasive mechanical ventilation support was extubated |  |
| Date ECMO support |  |
| Type of ECMO | 〇Vena-venous(V-V)  〇Vena-arterial(V-A)  〇Vena-arterial-venous(V-A-V) |
| Was the ECMO successfully decannulated (removed) during the hospitalization?  If the ECMO was decannulated for palliative purposes, select "no" | 〇Yes  〇No |
| Date ECMO was decannulated |  |
| Date CRRT support |  |
| Initial mode of CRRT | 〇Continuous(CRRT)-24h/day  〇Continuous(CRRT)-12h/day or less  〇Intermittent hemodialysis  〇Peritoneal dialysis  〇Other |
| Date CRRT was discontinued |  |
| Enter other mechanical support |  |
| **Labs** |  |
| **Blood routine test** |  |
| Red blood cell count |  |
| Hemoglobin |  |
| Hematocrit |  |
| Mean corpuscular hemoglobin concentration |  |
| Coefficient of variation of red cell distribution width |  |
| White blood cell count |  |
| Neutrophil count |  |
| Neutrophil count |  |
| Lymphocyte count |  |
| Lymphocyte count |  |
| Monocyte count |  |
| Monocyte count |  |
| Eosinophil count |  |
| Eosinophil count |  |
| Basophil count |  |
| Basophil count |  |
| Platelet count |  |
| **Serum biochemistry test** |  |
| Total Bilirubin |  |
| Conjugated(direct) bilirubin |  |
| Total protein |  |
| Albumin protein |  |
| Globulin protein |  |
| A/G ratio |  |
| Alanine transaminase |  |
| Aspartate transaminase |  |
| Alkaline phosphatase |  |
| Gamma-Glutamyl transferase |  |
| Prealbumin protein |  |
| Cholinesterase |  |
| Blood urea nitrogen |  |
| Creatinine |  |
| Estimated glomerular filtration rate |  |
| L-lactate dehydrogenase |  |
| Hydroxybutyrate dehydrogenase |  |
| Creatine kinase |  |
| Creatine kinase-MB mass |  |
| Creatine kinase-MB activity |  |
| Myoglobin |  |
| Troponin I |  |
| B-type natriuretic peptide |  |
| Sodium |  |
| Potassium |  |
| Calcium |  |
| Magnesium |  |
| Blood glucose |  |
| Total cholesterol |  |
| Triglycerides |  |
| High-density lipoprotein |  |
| Low-density lipoprotein |  |
| Uric acid blood |  |
| **Coagulation function** |  |
| Prothrombin time |  |
| International normalized ratio |  |
| Activated partial thromboplastin time |  |
| Thrombin time |  |
| Plasma fibrinogen |  |
| Plasma D-dimer |  |
| Fibrin degradation products |  |
| **Cytokines and inflammatory factors** |  |
| High-sensitivity C-reactive protein |  |
| Procalcitonin |  |
| Serum amyloid A |  |
| Serum ferritin |  |
| Interleukin-6 |  |
| Interleukin-10 |  |
| ESR |  |
| Additional Notes (if any) |  |

### Medications and adjuvant therapy

| The questions below refer to the first day following admission in hospital, continue to enter data until discharged from the hospital or dead (whichever occurs first) | |
| --- | --- |
| Antibiotics, antivirals, antifungal, Anticoagulation, corticosteroids, Granulocyte colony-stimulating factor, and Chinese medicine received at any time within following admission in hospital (select all that apply) | 〇Antibiotics  〇Antivirals  〇Antifungal  〇Corticosteroids  〇Granulocyte colony-stimulating factor  〇Chinese medicine  〇Other |
| Type of Antibiotics (select all that apply) | 〇Vancomycin  〇Piperacillin-tazobactam (Zosyn)  〇Azithromycin  〇Cephalosporins  〇Quinolones  〇Other |
| Enter the date vancomycin was initiated |  |
| Enter the date piperacillin-tazobactam was initiated |  |
| Enter the date azithromycin was initiated |  |
| Enter the date cephalosporins was initiated |  |
| Enter the date quinolones was initiated |  |
| Enter the date other antibiotics was initiated |  |
| Type of Antiviral (select all that apply) | 〇Arbidol hydrochloride  〇Ribavirin  〇Interferon  〇Oseltamivir  〇Ganciclovir  〇Peramivir  〇Entecavir  〇Other |
| Enter the date Arbidol hydrochloride was initiated |  |
| Enter the date Ribavirin was initiated |  |
| Enter the date interferon was initiated |  |
| Enter the date Oseltamivir was initiated |  |
| Enter the date Ganciclovir was initiated |  |
| Enter the date peramivir was initiated |  |
| Enter the date entecavir was initiated |  |
| Enter the date other antiviral was initiated |  |
| Type of Antifungal (select all that apply) | 〇Voriconazole  〇Amphotericin B  〇Caspofungin  〇Other |
| Enter the date voriconazole was initiated |  |
| Enter the date amphotericin B was initiated |  |
| Enter the date caspofungin was initiated |  |
| Enter the date other antifungal was initiated |  |
| Type of corticosteroids (select all that apply) | 〇Long-acting  〇Mid-acting  〇Short-acting |
| Enter the date long-action corticosteroid was initiated |  |
| Enter the name and cumulative daily dose of the Decadron  For example, if a patient was started on  Decadron 5mg twice per day, enter, " Decadron 10 mg " |  |
| Enter the date med-action corticosteroid was initiated |  |
| Enter the name and cumulative daily dose of the corticosteroid  For example, if a patient was started on  methylprednisolone 125mg twice per day, enter, "methylprednisolone 250 mg" |  |
| Enter the date short-action corticosteroid was initiated |  |
| Enter the name and cumulative daily dose of the hydrocortisone  For example, if a patient was started on  hydrocortisone 100mg twice per day, enter, " methylprednisolone 200 mg" |  |
| Enter the date other corticosteroids was initiated |  |
| Enter the date Granulocyte colony-stimulating factor was initiated |  |
| Enter the name and initial cumulative daily dose of the Granulocyte colony-stimulating factor  For example, if a patient was started on  Granulocyte colony-stimulating factor 100ug once per day, enter, " Granulocyte colony-stimulating factor 100 mg" |  |
| Enter the date Chinese medicine was initiated |  |
| Therapeutic anticoagulation includes any of the following categories:  1)Continuous drips (infusions) of heparin, argatroban, or bivalirudin  2)Subcutaneous regimens:  Enoxaparin (Lovenox) 1mg/kg twice per day or Enoxaparin (Lovenox) 1.5mg/kg once per day or Dalteparin (Fragmin) 150-200 units/kg once per day or Dalteparin (Fragmin) 100 units/kg twice per day or Fondaparinux at doses of 5mg or more daily  3)Oral anticoagulants: warfarin (Coumadin), apixaban (Eliquis), rivaroxaban (Xarelto), edoxaban, or dabigatran (Pradaxa) | 〇Yes  〇No |
| Anticoagulation type | 〇Heparin drip  〇Therapeutic enoxaparin (Lovenox)  〇Bivalirudin (Angiomax)  〇Argatroban  〇Other |
| Enter the date therapeutic anticoagulation was started |  |
| Enter the indication(s) for anticoagulation (select all that apply) | 〇Atrial fibrillation/flutter  〇Acute coronary yndrome/myocardial infarctions  〇DVT/PE  〇For extracorporeal circuit (e.g.,  ECMO or CRRT)  〇Hypercoagulable state associated  with COVID-19  〇Other |

### Mortality, Length of Stay

| Discharged from the hospital? -If the patient died in the hospital, select "no" -Enter the date the patient was first discharged from the hospital, even if they were subsequently readmitted back to the hospital | 〇Yes  〇No |
| --- | --- |
| Date of discharged from the hospital |  |
| Hospital mortality status | 〇Survived and discharged from the  hospital  〇Died during hospitalization  〇Patient is still hospitalized at the  time of data entry |
| Date of death |  |
| Cause(s) of death (select all that apply) | 〇ARDS/respiratory failure  〇Heart failure  〇Septic shock  〇Kidney failure  〇Liver failure  〇Other |
| Enter the other cause(s) of death  -Do not simply write, "cardiopulmonary arrest". Rather, provide the immediate underlying cause(s) of death in addition to those already listed above (e.g., pulmonary embolism, stroke, etc.)  -Would also mention here if the patient needed life-sustaining therapy (e.g., mechanical ventilation, RRT) but that it was unavailable due to shortages |  |
| Date discharged from the hospital |  |

## Supplementary Follow-Up Studies

### Follow-up Questionnaire

| **Symptom** **Questionnaire** | |
| --- | --- |
| 1. Do you have any obvious discomfort since you were discharged from hospital? | If yes, please specify |
| 2. How would you comment your current health status? | 〇Same as prior to COVID-19  〇Often feel fatigue, and easier to get  tired after activity now than prior to  COVID-19  〇Better health condition than prior to  COVID-19 |
| 4. Do you have any of the following symptoms that are newly onset post COVID-19 and persistent? | 〇Fever  〇Chills  〇Headache or dizzy  〇Myalgias  〇Fatigue  〇Rhinorrhea  〇Sore throat  〇Dry cough  〇Expectoration  〇Hemoptysis  〇Chest congestion  〇Dyspneal  〇Nausea or vomiting  〇Abdominal pain  〇Diarrhea  〇Consciousness disorder  〇Alopecia |
| 7. How do you feel about your sense of smell compared with the status prior to COVID-19? | 〇Same as before  〇Worse than before  〇Better than before  〇Total loss |
| 8. How do you feel about your sense of taste compared with the status prior to COVID-19? | 〇Same as before  〇Worse than before  〇Better than before  〇Total loss |
| 9. How do you feel about your appetite compared with the status prior to COVID-19? | 〇Same as before  〇Worse than before  〇Better than before |
| 10. What do you think about your sleeping compared with the status prior to COVID-19? | 〇Same as before  〇Worse than before  〇Better than before |
| 11. How do you feel about your muscle strength compared with the status prior to COVID-19? | 〇Same as before  〇Worse than before  〇Better than before |
| 12. Have you experienced hair loss now compared with the status prior to COVID-19? | 〇No hair loss before or after COVID-19  〇Hair loss is same as before  〇Lose more hair than before  〇Lose less hair than before |
| Additional Notes (if any) |  |
| **Lung CT Questionnaire** | |
| Scans of lung CT after discharge | 〇Yes  〇No |
| Enter the number of Reexamination of lung CT after discharge | 〇0  〇1  〇2  〇3 or more |
| Enter the date of the last lung CT scans |  |
| CT findings of the last lung scan | 〇Normal  〇Ground-glass opacity  〇Irregular lines  〇Consolidation  〇Interlobular septal thickening  〇Subpleural line  〇Reticular pattern |
| Additional Notes (if any) |  |
| **Health-related quality of life Questionnaire** | |
| 1. How do you feel about your mobility compared with the status prior to COVID-19? | 〇No problems with walking around  〇Slight problems with walking around  〇Moderate problems with walking around  〇Severe problems with walking around  〇Unable to walk around |
| 2. How do you feel about your personal care compared with the status prior to COVID-19? | 〇No problems with washing or dressing  〇Slight problems with washing or dressing  〇Moderate problems with washing or dressing  〇Severe problems with washing or dressing  〇Unable to wash or dress |
| 3. How do you feel about your usual activities compared with the status prior to COVID-19? | 〇No problems with usual activities  〇Slight problems with usual activities  〇Moderate problems with usual activities  〇Severe problems with usual activities  〇Unable to do usual activities |
| 4. Do you feel anxiety or depression compared with the status prior to COVID-19? | 〇No anxiety/depression  〇Slight anxiety/depression  〇Moderate anxiety/depression  〇Severe anxiety/depression  〇Extreme anxiety/depression |
| Additional Notes (if any) |  |
